# Evaluating Beryllium Exposure Data

**DOI:** 10.1289/ehp.114-a213a

**Published:** 2006-04

**Authors:** Marc Kolanz

**Affiliations:** Brush Wellman, Inc., Cleveland, Ohio, E-mail: marc_kolanz@brushwellman.com

We read with great interest “Chronic Beryllium Disease and Sensitization at a Beryllium Processing Facility” (Rosenman et al. 2005). We wish to offer some observations that will broaden the context in which this article is understood.

I agree with the statement by Rosenman et al. (2005) that a limitation of the study is the uncertainty of the exposure estimates. In addition, many statements appear to be unsupported by the data provided. For example, the statement that “most time-weighted averages were below the [Occupational Safety and Health Administration] OSHA (2005) standard of 2 μg/m^3^” (Rosenman et al. 2005) is unsupported by the data in the tables. Table 11 demonstrates that > 91% of the cohort had average daily weighted average (DWA) exposures > 2 μg/m^3^. Table 12 presents only the highest exposures and shows 56% of the cohort members having exposures > 2 μg/m^3^ and all but two cohort members exposed to > 0.2 μg/m^3^. Rosenman et al. (2005) did not explain how the average exposures of the cohort exceed 2 μg/m^3^ at a rate greater than the peak exposures. This same mysterious artifact of average exposures exceeding peak exposures is also present in Tables 9 and 10. The exposure-estimating process used by these authors could have introduced an erroneous bias in the data set, which causes me to question the “Discussion” and the conclusions drawn from the data.

One point, unstated by Rosenman et al. (2005), is that the DWA represented the daily exposure based on data averaged over 3 months. This is not the same as taking a single 8-hr air sample on 1 day because the 3-month averaging of the task data does not reveal the daily up and down variation in the individual task sample results. The National Institute for Occupational Safety and Health (NIOSH) has found DWAs not comparable to the lapel sampling method used by OSHA to determine compliance with its permissible exposure limit (PEL) or other 8-hr occupational exposure limits (OELs) (Donaldson and Stringer 1976). In addition, the average exposures presented by Rosenman et al. (2005) in their tables are the result of a second averaging by whole year and finally a third averaging by years of work. Such triple averaging further reduces the standard deviation in the data set, which results in a failure to identify the true high and low ranges of daily exposure. The American Industrial Hygiene Association (AIHA) exposure assessment guide (Mulhausen and Damiano 1998) cautions against ignoring air sample results that comprise the upper tail of an individual’s exposure distribution, especially when comparing it to PELs and OELs.

The flame spectroscopy method of chemical analysis of beryllium used by Rosenman et al. (2005) during the data-collection period of this study had a detection limit of 0.1 μg/filter that translates to < 0.1 μg/m^3^ for any lower value. Therefore, Rosenman et al. (2005) cannot make any statements about exposures lower than this value.

Rosenman et al.’s (2005) description of missing and estimated data, the illogical peak versus average data results, the triple averaged DWA exposure estimates, and the limit of analytical detection of the sampling method all combine to make it likely that virtually all members of the study population experienced multiple days of exposure > 2 μg/m^3^, and hence the study cannot sustain conclusions about the degree of risk associated with lower levels of exposure. This conclusion is supported by the observation that the rates of chronic beryllium disease (CBD) and sensitization were constant across all the categories of exposure used by Rosenman et al. (2005).

Rosenman et al. (2005) made no recommendations regarding how to protect beryllium workers. We cannot change the past, but we can learn from it and change the future. There are two successful models of beryllium safety: one demonstrates effectiveness in preventing clinical CBD (Johnson et al. 2001), and one demonstrates prevention of beryllium sensitization (BeS) using the beryllium blood lymphocyte proliferation test as an index of BeS (Cummings K, unpublished data). Common to both models are *a*) organization and cleanliness of the workplace; *b*) control of the upper range of air level exposure using engineering and respiratory protection; *c*) control of beryllium migration from the work process to the worker, the work area, and outside the facility; *d*) detailed training of workers; and *e*) management and worker commitment to effective program implementation. In the facility studied by Rosenman et al. (2005), it is not clear that any of the above elements of a beryllium safety management plan were consistently accomplished. Although this is understandable, given prevalent scientific opinion at the time, going forward we should make every effort to effectively disseminate these demonstrated beryllium safety principles to the companies and workers using beryllium.
